# Validation of a Phase-Mass Characterization Concept and Interface for Acoustic Biosensors

**DOI:** 10.3390/s110504702

**Published:** 2011-04-28

**Authors:** Yeison Montagut, José V. García, Yolanda Jiménez, Carmen March, Ángel Montoya, Antonio Arnau

**Affiliations:** 1 Phenomena Wave Group, Electronic Engineering Department, Universitat Politècnica de València, Spain; E-Mails: yeimonfe@doctor.upv.es (Y.M.); jogarnar@upvnet.upv.es (J.V.G.); yojiji@eln.upv.es (Y.J.); 2 Inter-University Research Institute for Bioengineering and Human Centered Technology, Universitat Politècnica de València, Spain; E-Mails: cmarch@ginmuno.i3bh.es (C.M.); amontoya@ginmuno.i3bh.es (A.M.)

**Keywords:** acoustic biosensors, sensitivity, microbalance, high resolution, phase characterization, high fundamental frequency QCM

## Abstract

Acoustic wave resonator techniques are widely used in in-liquid biochemical applications. The main challenges remaining are the improvement of sensitivity and limit of detection, as well as multianalysis capabilities and reliability. The sensitivity improvement issue has been addressed by increasing the sensor frequency, using different techniques such as high fundamental frequency quartz crystal microbalances (QCMs), surface generated acoustic waves (SGAWs) and film bulk acoustic resonators (FBARs). However, this sensitivity improvement has not been completely matched in terms of limit of detection. The decrease on frequency stability due to the increase of the phase noise, particularly in oscillators, has made it impossible to increase the resolution. A new concept of sensor characterization at constant frequency has been recently proposed based on the phase/mass sensitivity equation: Δφ/Δ*m* ≈ −1/*m_L_*, where *m_L_* is the liquid mass perturbed by the resonator. The validation of the new concept is presented in this article. An immunosensor application for the detection of a low molecular weight pollutant, the insecticide carbaryl, has been chosen as a validation model.

## 1. Introduction

In the fields of analytical chemistry, medical diagnostics and biotechnology, there is an increasing demand for highly selective and sensitive analytical techniques which, optimally, allow in real-time direct monitoring with easy to use, reliable and miniaturized devices. Different sensing technologies are being used for biochemical sensors. Regarding the transducer mechanism, electrochemical, optical and acoustic wave sensing techniques have emerged as the most promising technologies [1].

Acoustic sensing has taken advantage of the progress made in the last decades in piezoelectric resonators for radio-frequency (RF) telecommunication technologies. The so-called gravimetric technique [2] has opened a great deal of applications in bio-chemical sensing in both gaseous and liquid media.

Many of the biochemical interactions can be evaluated and monitored in terms of mass transfer over the appropriate interface. This characteristic allows using the gravimetric techniques based on acoustic sensors for a label-free and a quantitative time-dependent detection. Acoustic sensor based techniques combine their direct detection, real-time monitoring and high sensitivity, with the selectivity provided by the appropriate sensor surface functionalization and bio-reagent selection (e.g., monoclonal antibody or hapten-conjugate). Additionally, the key measuring magnitude of acoustic wave devices is the frequency, or phase, of a signal which can be easily and precisely processed with very simple electronic devices; this provides high integration capability of the sensor device with the associated electronics and read-out electronic systems.

The classical quartz crystal microbalance (QCM) has been the most used acoustic device for sensor applications; however, other acoustic devices have been, and are being used for the implementation of nano-gravimetric techniques in biosensor applications. An overview of the different techniques used in the implementation of acoustic biosensors could be very useful for three reasons: first because it gives a complete updated sight of the acoustic techniques currently used in biosensors, second because some of the challenges remaining for acoustic biosensors are mostly common to all the acoustic devices, and third because the new aspects presented in this article, in particular for QCM, can be considered for the other devices as well. With this purpose, a brief description of the state of the art of the different acoustic techniques used in biosensors is included next.

### 1.1. Bulk Acoustic Wave Devices (BAW)

**Figure 1.**
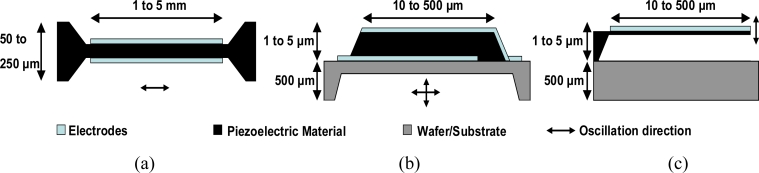
Bulk acoustic devices: **(a)** QCM, **(b)** FBAR and **(c)** Cantilevers.

In bulk acoustic wave (BAW) devices, waves travelling or standing in the bulk of the material are excited, through the piezoelectric or capacitive effects, by using electrodes on which an alternative voltage is applied. The three important BAW devices are quartz crystal microbalances (QCMs), film bulk acoustic resonators (FBARs) and cantilevers. Figure 1 shows their basic structure and typical dimensions. Because the vibrating mode of cantilevers is not suited for operation in liquids due to the high damping, we will focus our discussion on QCM and FBAR devices.

#### 1.1.1. QCM for Biosensing Applications

In the classical QCM configuration shear waves are excited by a sinusoidal voltage applied to the electrodes [see Figure 1(a)], which makes operation in liquids viable [3]. The theoretical absolute frequency/mass sensitivity is proportional to the square of the resonant frequency according to the following expression [2]:

(1) $$S_{a}=\frac{\Delta f}{\Delta m}=-\frac{2}{\rho \nu }\frac{f_{n}^{2}}{n}$$

where Δ*f* is the resonance frequency shift, Δ*m* is the surface mass density change on the active sensor surface, *ρ* is the quartz density, *v* the propagation velocity of the wave in the crystal, *f_n_* is the frequency of the selected harmonic resonant mode and *n* is the harmonic number (*n* = 1 for the fundamental mode).

The so obtained theoretical mass sensitivity is right only under ideal conditions [4–7]. Absolute sensitivities of a 30 MHz QCM reach 2 Hz·cm^2^·ng^−1^, with typical mass resolutions around 10 ng·cm^−2^ [8]. An improvement in the resolution down to 1 ng·cm^−2^ by optimizing the characterization electronic interface as well as the fluidic system seems possible. This technique has been extensively used in the literature for the monitoring of many detection processes in biochemistry and biotechnology [9–17].

Despite the extensive use of QCM technology, some challenges such as the improvement of the sensitivity and the limit of detection in high fundamental frequency QCM remain unsolved; recently, an electrodeless QCM biosensor for 170 MHz fundamental frequency, with a sensitivity of 67 Hz·cm^−2^·ng^−1^, has been reported [18]; this shows that the classical QCM technique still remains a promising technique. Once these aspects are solved, the next challenge would be the integration capability; in this sense, commercial QCM systems are mostly based on single element sensors, or on multi-channel systems composed by several single element sensors [19]. They are currently expensive, mainly because their manufacturing is complex, especially for high frequencies, and their application for sensor arrays is difficult due to lack of integration capability and appropriate characterization interface. Some of these shortcomings could be overcome with the appearance of film bulk acoustic resonators (FBARs).

#### 1.1.2. FBAR Devices for Biosensing Applications

A typical film bulk acoustic resonator (FBAR) consists of a piezoelectric thin film (such as ZnO or AlN) sandwiched between two metal layers. A membrane FBAR is shown in Figure 1(b). In the past few years, FBARs on silicon substrates have been taken into account for filter applications in RF devices [20]. Gabl *et al*. were the first to consider FBARs for gravimetric bio-chemical sensing applications [21]. They basically operate like QCMs; however, typical thicknesses for the piezoelectric thin film are between 100 nm and a few μm, allowing FBARs to easily reach resonance frequencies in the GHz range. The main advantage of FBAR technology is its integration compatibility with CMOS technologies, which is a prerequisite for fabrication of sensors and sensor arrays integrated with electronics, and hence reduces the cost of miniature sensor systems.

According to Equation (1), FBAR devices could provide sensitivities higher than QCMs, due to the higher resonance frequency of those devices; however, this higher sensitivity does not necessarily mean that a higher mass resolution is achieved. Effectively, thin film electroacoustic technology has made possible the fabrication of quasi-shear mode thin film bulk acoustic resonators (FBARs), operating with a sufficient electromechanical coupling to be used in liquid media at 1–2 GHz [22,23]; however, the boundary conditions, due to the higher frequency and the smaller size of the resonator, have a much stronger effect on FBAR performance than on the QCM response. A higher mass sensitivity is attained, but with an increased noise level as well, thus moderating the gain in resolution [24,25]. So far only publications on network analyzers based on FBAR sensor measurements have been published in the literature, which show that the FBAR mass resolution is very similar if not better than that of oscillator based on QCM sensors [24–27]. The first shear mode FBAR biosensor system working in a liquid environment was reported in 2006 [26]; the device had a mass sensitivity of 585 Hz·cm^2^·ng^−1^ and a limit of detection of 2.3 ng·cm^−2^, already better than that obtained with QCM (5.0 ng·cm^−2^) for the same antigen/antibody recognition measurements. However, these results have been compared with typical 10 MHz QCM sensors; therefore high fundamental frequency QCM sensors working, for instance, at 170 MHz could have much higher resolution than the reported FBAR sensors [18]. In 2009 a FBAR for the label-free biosensing of DNA attached on functionalized gold surfaces was reported [28]. The sensor operated at about 800 MHz, had a mass sensitivity of about 2,000 Hz·cm^2^·ng^−1^ and a minimum detectable mass of about 1 ng·cm^−2^. However, studies that focus exclusively on the mass sensitivity do not provide a comprehensive view of the major factors influencing the mass resolution. For instance in FBAR sensors, in contrast to the conventional QCMs, the thickness of the electrodes is comparable to that of the piezoelectric film and hence cannot be neglected. Therefore, the FBAR must be considered like a multilayer structure, where the acoustic path includes the piezoelectric film as well as an acoustically “dead” material (e.g., electrodes) and additional layers such as for instance Au, which is commonly used as a suitable surface for various biochemical applications, or SiO_2_ which is also used for temperature compensation [29]. In general there is a set of factors such as loss mechanisms, multilayer effects, lateral structure, spurious modes, *etc*. which affect the quality factor of a FBAR sensor and hence must be considered.

Another approach used to get higher mass sensitivities by increasing the frequency is through using surface generated acoustic wave devices (SGAWs).

### 1.2. Surface Generated Acoustic Wave Devices (SGAW)

**Figure 2.**
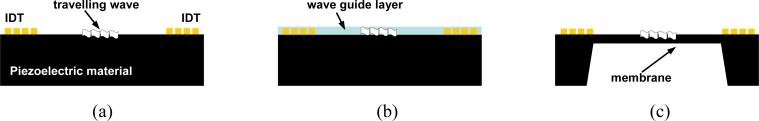
Different types of SGAW devices: **(a)** typical SAW configuration, **(b)** Love-wave SGAW device and **(c)** flexural plate SGAW device.

SGAW devices have been used as chemical sensors in both gaseous and liquid media. By applying a RF signal to an input port made with interdigital electrodes (IDTs), a mechanical acoustic wave is launched into the piezoelectric material due to the inverse piezoelectric phenomenon, and propagates through the substrate reaching an output IDT. The separation between the IDTs defines the sensing area where biochemical interactions at the sensor surface provide changes in the properties of the acoustic wave (wave propagation velocity, amplitude or resonant frequency) [30]. Figure 2 shows a schematic view of different SGAW devices.

In SGAW devices the acoustic wave propagates, guided or unguided, along a single surface of the substrate. SGAW devices are able to operate, without compromising the fragility of the device, at higher frequencies than QCMs [31], thus increasing the sensitivity [32–34]. Many SGAW devices working in shear horizontal mode have been reported as more sensitive than the typical QCM-based devices [35]. In most cases, Love-wave devices are preferred; they operate in the SH wave mode with the acoustic energy trapped within a thin guiding layer (typically submicrometer), which enhances the sensitivity by more than one order of magnitude in comparison with a different SAW device [36,37]. In addition, the wave guide layer in the Love mode biosensor could, in principle, also protect and insulate the IDTs from the liquid media which might otherwise be detrimental to the electrode. Therefore, they are frequently utilized to perform bio-sensing in liquid conditions [38–46], arising as the most promising SGAW device for this purpose [43,47].

The mass sensitivity of LW sensors has been evaluated [48–50]. Kalantar and coworkers reported a sensitivity of 95 Hz·cm^2^·ng^−1^ for a 100 MHz Love mode sensor, which is much better than the typical values reported for low frequency QCM technology [51], although similar to those reported for high fundamental frequency QCM—67 Hz·cm^−2^·ng^−1^ [18]. However, Moll and coworkers reported a LOD for a Love sensor of 400 ng·cm^−2^ [43]; although this reported sensitivity can depend on other factors apart from the device itself, this reveals once again that an increase in the sensitivity does not mean, necessarily, an increase in the resolution. Moreover, in spite of the initial advantage of the guiding layer for isolating the IDTs, in real practice the capacitive coupling between the IDTs due to the higher permittivity of the liquid makes necessary to avoid the contact of the liquid with the guiding layer just over IDTs, at the same time that it is necessary to allow the contact of the central area between the IDTs with the liquid medium. This increases the complexity of the design and practical implementation of the flow cell for LW acoustic devices; this is one of the reasons why there are very few commercial microgravimetric systems based on LW-devices for in-liquid applications.

Consequently, although acoustic techniques have been improved in terms of robustness and reliability and allow the measurement of molecular interactions in real time, some important challenges are still unresolved: the improvement of the sensitivity, but with the aim of getting a higher mass resolution; and the integration capability, which make the simultaneous characterization of multiple sensors and multi-analysis detection possible.

This article is focused on QCM technology applied to biosensors. A new characterization interface based on the phase-shift characterization concept introduced elsewhere [52], is compared with the classical characterization method based on resonance frequency-shift using an improved oscillator configuration. With this purpose in mind, a QCM immunosensor for the detection of a low molecular weight pollutant, the insecticide carbaryl, described elsewhere [53], has been used as a validation model. The obtained results validate the new sensor characterization concept and system as a new QCM characterization technique. Moreover, this technique offers the opportunity of undertaking the remaining challenges in the acoustic biosensor technologies: (1) improvement in the sensitivity and limit of detection by working with very high frequency QCM sensors; and (2) the possibility to easily implement a QCM sensor array system with a high level of integration capability. These important aspects will be clarified later on.

## 2. Phase-Mass Characterization Concept for In-Liquid High Resolution QCM Applications

Following a similar mathematical development described elsewhere [52], the next generalized approximated equation for the phase-shift of a signal, of constant frequency very close to the motional series resonant frequency of the resonator-sensor, to small changes both in the coating mass and liquid properties, is found:

(2) $$\Delta \varphi (\text{rad})=-\frac{1}{m_{q}+m_{L}}\Delta m_{c}-\frac{m_{q}-m_{c}}{{\left(m_{q}+m_{L}\right)}^{2}}\Delta m_{L}$$

where *m_q_* = *η_q_π/*2*v_q_*, being *η_q_* the effective quartz viscosity and *v_q_* the wave propagation speed in the quartz; *m_c_* is the surface mass density of the coating and *m_L_* = *ρ_L_δ_L_*/2, where *ρ_L_* and *δ_L_* are, respectively, the liquid density and the wave penetration depth of the acoustic wave in the liquid.

In biosensor applications *m_L_* can be assumed to be constant and the previous equation reduces to:

(3) $$\Delta \varphi (\text{rad})=-\frac{\Delta m_{c}}{m_{q}+m_{L}}$$

which was already obtained for small coating mass changes [52].

For most in-liquid QCM applications *m_q_* ≪ *m_L_*, and the former equation can thus be approximated to:

(4) $$\Delta m_{\text{min}}\approx -m_{L}\;\Delta \varphi_{\text{min}}$$

This equation establishes the mathematical base for the phase-shift characterization concept. Currently, all the QCM sensor characterization techniques provide, among other relevant parameters, the resonance frequency shift of the sensor [54–56]: network or impedance analysis is used to sweep the resonance frequency range of the resonator and to determine the maximum conductance frequency [57,58], which is almost equivalent to the motional series resonance frequency of the resonator-sensor. Impulse excitation and decay method techniques, whose major representative is the QCM-D system, are used to determine the series-resonance or the parallel-resonance frequency depending on the measuring set-up [59,60]. Oscillator techniques are used for continuous monitoring of a frequency which corresponds to a specific phase shift of the sensor in the resonance bandwidth [61–65]; this frequency can be used, in many applications, as a reference for the resonance frequency of the sensor; and the lock-in techniques, which can be considered as sophisticated oscillators, are designed for a continuous monitoring of the motional series resonance frequency, or the maximum conductance frequency, of the resonator-sensor [66–72]. To assure that the frequency shift is the only parameter of interest, a second parameter is important, for instance in piezoelectric biosensors, which provide information of the constancy of the properties of the liquid medium; this parameter depends on the characterization system, being: the maximum conductance or the conductance bandwidth in impedance analysis, the dissipation factor in decay methods and a voltage associated with the sensor damping in oscillator techniques.

The most accurate characterization methods are those which interrogate the sensor with a very low noise external source; this is the case of impedance or network analyzers and decay based methods. Phase-locked loop architectures could be ascribed in this category as well, as long as the voltage controlled oscillator included in the loop is based on a very low noise source; however this is not usually the case, and the final frequency stability is mainly provided by the sensor quality factor, having, in principle, the same stability problems seen in typical oscillators. Consequently, phase-locked loop techniques should be considered more like very sophisticated oscillators, which have the advantage of being easily calibrated at the desired oscillation phase condition, and therefore it is better to consider them in a different category.

Impedance analysis is routinely used for sensor characterization, mainly in very high frequency applications. Decay techniques can be used for relatively high frequency sensors, a frequency limit of 70 MHz is specified for the Q-Sense QCM-D system. These techniques interrogate the sensor with a burst signal of appropriate frequency and then the decay shape must be registered at regular intervals; by appropriate signal processing the resonance frequency and the dissipation factor are obtained. To improve the stability and reduce the noise level, averaging of the measured data is necessary, mainly at very high frequencies [18]; therefore decay methods, although being faster than impedance analyzers for sensor characterization, are not appropriate for very fast sensor frequency changes as seen, for instance, in ac-electrogravimetry applications [73].

Consequently, for high fundamental frequency QCM applications, oscillators have been mostly used due to the low cost of their circuitry, as well as their integration and continuous monitoring capability. However, in spite of the efforts carried out to build oscillator configurations suitable for in-liquid applications [74–81], the poor stability of high frequency QCM systems based on oscillators has prevented the increase of resolution despite the higher sensitivity reported [82–85]. The main reasons are discussed elsewhere [52,86].

**Figure 3.**
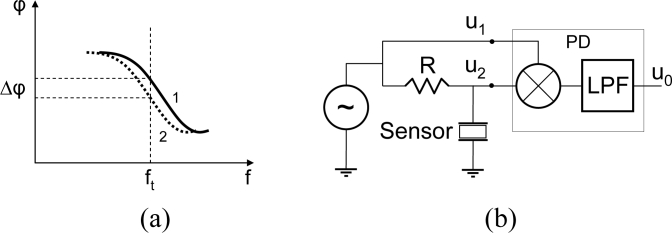
**(a)** Description of the phase approach and **(b)** Simple implementation.

By keeping in mind the previous considerations, a different approach was recently proposed [52]: taking into account that the expected frequency shifts in QCM biosensors are very small, it could be possible to interrogate the sensor with an appropriate constant frequency signal, in the sensor resonance bandwidth, and then to measure the change in the phase response of the sensor while maintaining the frequency of the testing signal in the resonance bandwidth; Figure 3(a) depicts this idea. A similar approach has been already applied by some authors [87,88]. The advantage of this approach is that the sensor is interrogated with an external source which can be designed to be very stable, and with extremely low phase and frequency noises, even at very high frequencies. Additionally, the phase-shift changes can be continuously monitored even for very fast changes in the sensor response. Moreover, a very simple circuit can be used for the phase-mass characterization approach, as depicted in Figure 3(b), where a mixer based phase detector is used. A more practical implementation will be discussed in the next section.

It should be pointed out that in the phase-shift technique the testing frequency must be close to the motional series resonant frequency, otherwise the previous equations are not valid. The same happens with the frequency-shift technique. Effectively, from theory only shifts of the motional series resonant frequency (frequency at maximum conductance) reflect the correct surface mass change of the coating, assuming the properties of the liquid are constant; any other frequency is influenced by energy dissipation. Therefore when changes in the liquid occur, an additional magnitude is necessary to discriminate the mass change due to the coating from the mass change effect due to the change in the liquid properties. This also happens in the phase-shift technique, and an additional magnitude, for example the power ratio between the signals u_1_ and u_2_ in Figure 3(b), could be used for this purpose. On the other hand, it should be pointed out as well that the phase-shift technique is useful for high resolution QCM applications, where tiny frequency changes of the resonant frequency are expected, and the testing frequency remains close to the maximum conductance frequency.

## 3. Phase-Mass Characterization Interface for In-Liquid High Resolution QCM Applications

**Figure 4.**
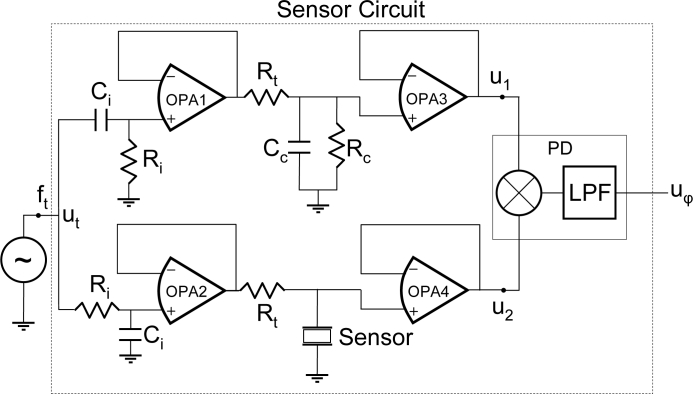
Schematics of the interface system for the sensor phase characterization.

The sensor circuit depicted in Figure 4 has been implemented to validate the new characterization concept. Two parallel branches form a differential circuit. Because the testing signal *u_t_* has constant frequency *f_t_*, the only element in the circuit which contributes to a change in the phase shift between the reference signal *u_1_* and the signal *u_2_*, is the change in the phase-frequency response due to the sensor perturbation. Therefore, this phase-shift can be continuously monitored by a phase-detector. The mixer and the low-pass filter (LPF), connected in series behind the signals *u_1_* and *u_2_*, act as a phase detector (PD) for small phase-shifts around 90° between the input signals [52]. Thus, for a proper operation it is convenient to phase-shift, in advance, the testing signals in each branch of the sensor circuit 90°; for this purpose the networks formed by *R_i_* and *C_i_* at the inputs of the sensor circuit have been included. The phase-shifting networks formed by *R_i_* and *C_i_* must be coherently designed with the resonant frequency of the sensor in order to obtain two signals 90° phase-shifted and of similar amplitude.

Wide bandwidth operational amplifiers OPA1–4 are used to isolate the sensor and the reference network *R_c_*-*C_c_* from the rest of the circuit. At motional series resonance frequency (MSRF), the sensor is reduced to a motional resistance, R_m_, in parallel with the so-called static capacitance C_0_; therefore for optimum operation it is convenient to select *R_c_* and *C_c_* similar to *R_m_* and *C*_0_, respectively. Effectively, under these conditions, and at the MSRF of the sensor, the voltage u_φ_ corresponding to the phase-shift should be zero; this provides a way to calibrate the system.

Additionally, far from resonance the sensor behaves like the parallel capacitance *C*_0_, and the network formed by the resistance *R_t_* and the sensor acts like a low-pass filter *R_t_*-*C*_0_ of very high cut-off frequency around several megahertz. Consequently slow phase noises in the input testing signal are transferred equally to both branches and are eliminated by the phase differential detection, and then the stability is improved.

## 4. Experimental Validation of the Phase-Mass Characterization Concept and Interface

A comparison between the classical technique of frequency shift monitoring, based on an improved version of a balanced bridge oscillator proposed elsewhere [54,89], and the new one based on the phase-shift monitoring concept, is presented next to validate the proposed technique. Only with this purpose in mind, a piezoelectric immunosensor for the detection of the pesticide carbaryl, has been developed as a validation model. It is important to clarify that both techniques were compared under the same experimental conditions; it means that the different electronic circuitries were connected to the same sensor with the same experimental set-up.

### 4.1. Experimental Methodology

**Figure 5.**
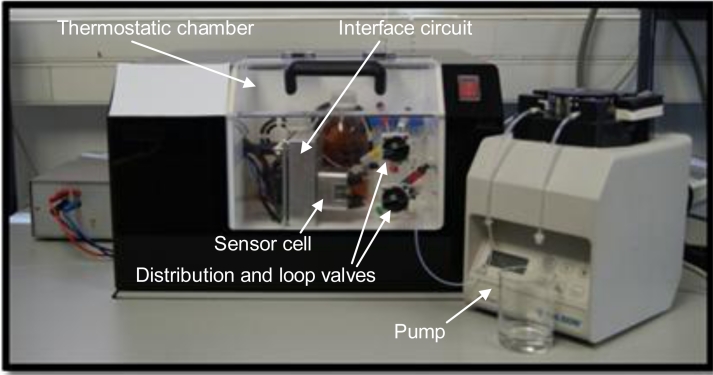
Experimental Setup.

AT-cut quartz crystals with gold electrodes (10 MHz, International Crystal Manufacturing) were functionalized by immobilizing BSA-CNH carbaryl hapten conjugate on the sensor surface through the formation of a thioctic acid self-assembled monolayer [53]. The crystal was placed in a custom-made flow cell and included in a flow-through setup, controlled by a peristaltic pump (Minipuls 3, Gilson), with the injection loop and solutions at the input of the flow cell exchanged by manual Rheodyne valves (models 5020 and 5011, Supelco). The whole fluidic system and the sensor characterization circuit with the sensor cell were placed in a custom made thermostatic chamber and all the experiments were performed at 25 °C ± 0.1 °C. To avoid unwanted disturbances the chamber was placed on an anti-vibration table. A RF signal generator model HP8664A (Hewlett-Packard) generated the signal applied to the circuit and the voltage variations related to the phase shift were measured with a digital multimeter HP 34401A (Agilent) and sent to a PC via GPIB bus. The experimental set-up is presented in Figure 5.

The immunoassay developed to determine carbaryl was an inhibition test based on the conjugate coated format, in which the hapten-conjugate was immobilized on the sensor surface. A fixed amount of the respective monoclonal antibody was mixed with standard solutions of the analyte and pumped over the sensor surface. Since the analyte inhibits antibody binding to the respective immobilized conjugate, increasing concentrations of analyte will reduce the phase shift induced on the piezoelectric sensor and the corresponding demodulated voltage.

Different standard concentrations of carbaryl were prepared by serial dilutions in PBS, from a 1 mM stock solution in dimethylformamide at −20 °C. The standards were mixed with a fixed concentration of the monoclonal antibody LIB-CNH45 (from I3BH-UPV, [90]) in PBS. Analyte-antibody solutions were incubated for one hour at chamber temperature (25 °C) and then injected onto the sensor surface. The phase-shift was monitored in real-time for each analyte concentration. For each assay, after stabilization of the initial signal at a flow rate of 30 μL/min for 2 min, the sample (250 μL) was injected and the immunoreaction was monitored for 12 min. Once each assay was finished, regeneration of the sensing surface was performed using diluted hydrochloric acid (0.1 M HCl) at a flow rate of 280 μL/min for 4 min to break the antibody-hapten linkage. After the regeneration, buffer solution was flowed again for 2 min at the same flow rate.

### 4.2. Results and Discussion

**Figure 6.**
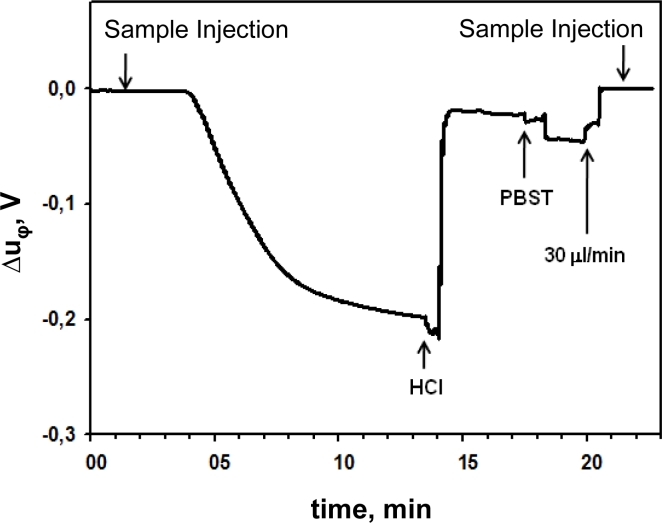
Real-time signal obtained for piezoelectric immunosensor with the phase-shift characterization system.

Figure 6 shows the typical real-time signal obtained in the immunoassay developed for the detection of carbaryl with the phase shift concept. The voltage Δu_φ_, associated with the phase-shift, decays as soon as the molecular interaction occurs after the sample injection; a regeneration step is performed by consecutive injections of HCl and phosphate buffered saline—Tween 20 (PBST) at appropriate concentrations, reaching the initial base-line.

**Figure 7.**
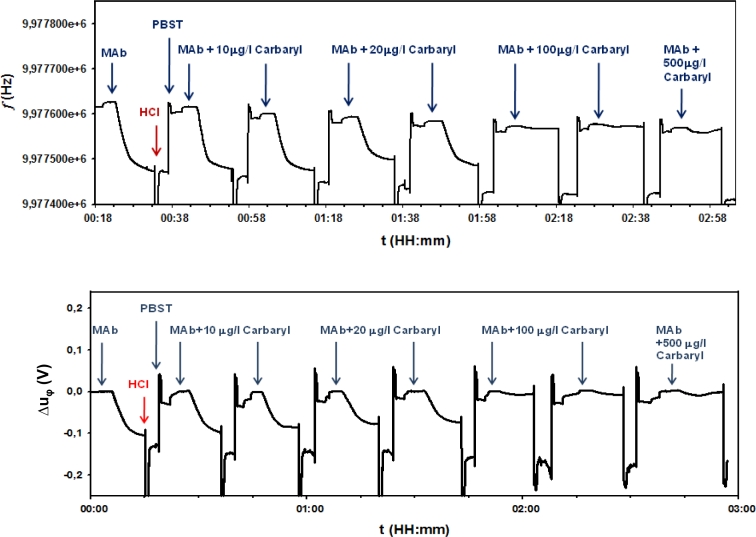
Real time piezoelectric immunosensor response to different concentrations of analyte: with the balanced-bridge oscillator (upper panel), and with the phase-shift characterization system (lower panel).

A similar behavior is obtained when the resonant frequency shift is monitored. Figure 7 shows a comparison between the real-time signals obtained in the piezoelectric immunosensor for the same experiment with the frequency-shift and phase-shift monitoring systems. During the experiments, different concentrations of pesticide in the sample were tested after cyclic regeneration steps as explained. Only a representative part of the signals obtained in the immunoassay, corresponding to concentrations of antibody-analyte of 10, 20, 100 and 500 μg/L is shown in Figure 7.

**Figure 8.**
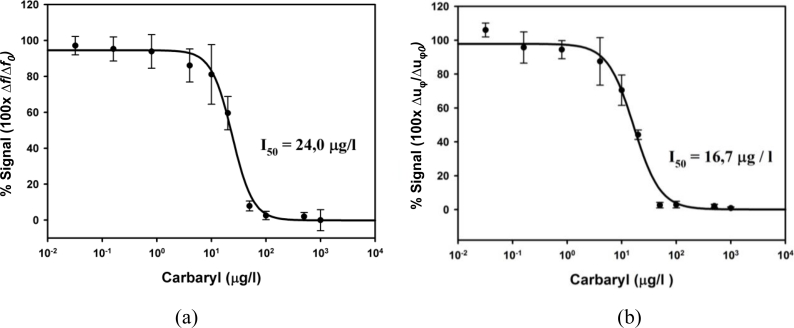
Average standard curve for the carbaryl piezoelectric immunosensor: **(a)** by using the classical frequency-shift characterization with the balanced-bridge oscillator proposed elsewhere [54,89], and **(b)** by using the phase-shift characterization method and interface.

A representative standard curve (Figure 8), for each system, was finally obtained by averaging three individual standard curves starting from samples that were run at least in duplicate. In Figure 8 the decrease of the phase voltage has been normalized and represented as a percentage of the maximum decrement obtained (100 × Δu_φ_/Δu_φ0_, being Δu_φ_ the voltage variation of each sample and Δu_φ0_ the variation for the zero analyte concentration sample, which provides maximum signal). The experimental points were fitted to a four-parameter logistic equation [53], then showing the typical decreasing sigmoidal shape of binding inhibition immunoassays.

The relevant parameters of interest of the immunosensor: *I_50_* value, defined as the analyte concentration which provides 50% inhibition of the maximum signal, and typically related to the sensitivity; the limit of detection (LOD), defined as the pesticide concentration that provides 90% of the maximum signal (*I_90_* value), and related to the resolution; and the quantification range, defined as the working range in which the signal inhibition is linear, are summarized, for the two characterization methods and interfaces in Table 1; previously reported results based on the frequency-shift monitoring with a different commercial electronic interface are included as well for comparison [53].

As it can be observed, both the sensitivity and limit of detection of the developed immunosensor were of the same order of magnitude for the different characterization interfaces. These results validate the new characterization concept and the proposed interface. An improvement trend of the analytical parameters (I_50_ and LOD), due to the small reduction of the noise in the new system, is observed as well. Effectively, the noise level in the oscillator technique was of 2 Hz for a maximum signal of 137 Hz, while for the phase-shift interface was of 1 mV for a maximum signal of 200 mV; this indicates an improvement of three times in the noise in relation to the maximum signal. It is important to notice that the improvement trend has been got even with low frequency sensors (10 MHz), where all the electronic components and circuits used have a very good performance. This noise reduction has been partially transferred into a small improvement in the limit of detection, which must only be considered as a trend. Perhaps a further improvement in the resolution could have been obtained by optimizing the biochemical parameters, but this was not the main purpose of this work; moreover, to work in the optimization of the biochemical parameters would only be worth it when an important increase in the maximum signal is obtained. For that, a real improvement in the sensitivity by using much higher fundamental frequency resonators is necessary. However, the validation of the phase-shift technique in QCM biosensor applications, even for relatively low frequency sensors, is important because it opens new strategies for monitoring very high frequency QCM sensors, in those applications in which high resolution is necessary. Moreover, differential phase systems are typically used for measuring the noise level in high frequency oscillator configurations; consequently, the phase shift method can be directly implemented at very high frequencies. Therefore additional experiments with very high frequency sensors can be readily performed to demonstrate whether an increase in the sensitivity would directly become into a resolution increase or not.

## 5. Conclusions

The new method for QCM biosensor characterization, based on the monitoring of the phase-shift experienced by a signal of constant frequency in the resonant bandwidth of the sensor, has been validated under real-experimental conditions, and compared with classical interface techniques. An improvement trend, both in sensitivity and limit of detection, is observed, even for relative low frequency resonators (10 MHz), due to the signal to noise ratio improvement. Moreover, the new characterization system, particularly useful for biosensor applications, has special advantages which make it ideal for addressing the remaining challenges in high resolution QCM applications: (a) the sensor is passively interrogated with an external source, which can be designed with high frequency stability and very low phase noise, even at very high frequencies, (b) the open loop configuration, in contrast to the typical feedback configuration of the oscillator, allows a straightforward noise analysis and minimization, simplifying the design and implementation of the electronics, and (c) sensors working at the same fundamental resonance frequency could be characterized, in principle, with one source, only repeating the sensor circuit depicted in Figure 4 which has high integration capability. This opens the possibility of working with sensor arrays for multianalysis detection. Following the results presented here, the next step is to perform experiments with high fundamental frequency QCM resonators.

## Figures and Tables

**Table 1.** Comparative results obtained for the QCM immunosensor using different electronic characterization techniques.

	
	**Phase Shift Method**	**Oscillator [89]**	**[53]**
Sensitivity *I_50_* (μg/L)	16.7	24.0	30.0
L.O.D. *I_90_* (μg/L)	4.0	6.5	11.0
Linear Range (μg/L)	7–35	11–42	15–53
